# Failure analysis and prediction of roof instability in end face under repeated mining using early warning system

**DOI:** 10.1038/s41598-023-35685-5

**Published:** 2023-05-30

**Authors:** Fei Li, Dezhong Kong, Qiang Li, Yuqi Shang, Zhanbo Cheng, Liuquan He

**Affiliations:** 1 Mining College, Guizhou University, Guiyang, 550025 China; 2 School of Energy and Mining Engineering, China University of Mining and Technology-Beijing, Beijing, 100083 China; 3 School of Civil and Environmental Engineering, Nanyang Technological University, Nanyang, 639798 Singapore; 4 State Key Laboratory of Public Big Data, Guizhou University, Guiyang, 550025 Guizhou China

**Keywords:** Engineering, Coal, Software

## Abstract

The overlying strata of the lower coal seam is easy to be collapsed causing the roof caving accident at the end face of the mining working face under repeated mining in close-distance coal seams. In order to predict the roof instability of the end face, the mechanical model of the granular arch structure is established in this study to further analyze its main influencing factors. The results show that the mining height of the working face, the advancing speed, the distance of coal seams, the tip-to-face distance, the strength of the surrounding rock and the support setting the load of the support are the main influencing factors on the roof caving of the end face. Subsequently, the prediction model of roof instability in the end face under repeated mining is constructed through the radial basis function neural network (RBFNN) and the above main influencing factors are regarded as input layer indexes. Meanwhile, the roof subsidence, coal wall deformation and support load are determined as the output layer indexes. The predicted results are closer to the results of sample tests. Finally, the early warning system, including monitoring and early warning, data query, emergency management, user management, and system settings, is designed to monitor roof conditions of the end face and timely warn the roof accidents. The field application proves that the system has good practical value, which is of great significance to intelligent prediction of coal mine stope disaster and prevent the end face roof disaster under repeated mining and. This will promote the safe and efficient construction of coal mine production.

## Introduction

Close-distance coal seams are common coal mining conditions in China's coal mines. In the downward mining process, the roof of the lower coal seam is affected by repeated mining along with the reduction of its bearing capacity^1,2^. In addition, the loose gangue falling from the goaf side after the mining of the upper coal seam can be covered on the roof of the lower coal seam, which leads to the roof of the lower coal seam prone to collapse or even the occurrence of large-scale end face roof caving accidents, thus affecting the normal production of coal mines^3–6^. The problem of end face roof caving under repeated mining conditions is more serious in the mining of close-distance coal seams in southwest China. The internal mechanism and early warning of end face roof instability under repeated mining of close-distance coal seams have their particularity. In China, with the introduction of the concepts of 'smart coal mine' and 'intelligent unmanned mining technology', the status of roof disaster warning and prediction in coal scientific mining is getting higher and higher^7,8^. How to accurately predict the stability of end face roof of close-distance coal seams under repeated mining has become an important research content of coal mining under complex occurrence conditions.

Studying the roof of the working face of the underlying coal seam in the close-distance coal seams is to study the surrounding rock of the repeated mining of the coal seams. In view of the research on the theory of surrounding rock failure under repeated mining of the coal seams, Li et al.^9^ combined the two research methods of physical similarity simulation and numerical simulation, and clarified that the process of roof failure of the underlying coal seam in the repeated mining of the coal seams is a process of filling the goaf with the natural caving method of coal gangue after the mining of the overlying coal seam, the integrity of the main roof of the underlying coal seam is destroyed, and the rock strata between the coal seams are broken and collapsed. Xie et al.^10^ combined the theory of floor damage depth with the theory of rock damage, calculated the first weighting interval and periodic weighting interval of the damaged floor strata (i.e., the main roof of the underlying coal seam) after the overlying coal seam mining, and clarified the instability conditions of the internal key blocks after the rock damage. Experts and scholars in the mining industry have conducted a lot of explorations on the factors affecting the stability of end face roof. He et al.^11^ analyzed the effect of support in controlling roof fall and rib spalling through setting up a caving arch mechanical model of cataclastic coal and rock mass on fully mechanized caving face and determined the key parameters of support by numerical simulation. Ju et al.^12^ clarified the difference of end face roof caving between great mining height and low mining height through similar simulation experiment and theoretical analysis, and pointed out that the reason for end face roof caving in great mining height working face even under the conditions of complete roof and unobvious rib spalling of working face was that it was difficult to form a self-stable structure due to the lack of lateral force after the block of key layer in the main roof was broken. Once the support supporting force was insufficient, it would lead to the through end face roof caving accident in mining working face. Guo et al.^13^ through the method of theoretical analysis, analyzed the instability characteristics of the coal wall and the immediate roof of the upward mining face, established the mechanical relationship model to maintain the stability of the coal wall and the end face roof, expounded the method of controlling the stability of the end face roof under different upward mining angles, that is, according to different situations to determine the reasonable support setting load and the front angle of column. Kong et al.^14^ took a coal mine in Guizhou Province of China as the research object. By establishing a physical model, he studied the roof structure and roof pressure law under repeated mining conditions, and determined that the main influencing factors of end face roof instability were support working resistance, tip-to-face distance, and surrounding rock strength. Using UDEC numerical simulation software, he pointed out that the higher the strength of roof and surrounding rock, and the smaller the tip-to-face distance, the better the stability of end face roof. Li et al.^15,16^ analyzed the different influencing factors of roof collapse at the end of face in repeated mining, and clarified that the influence degree of different factors from large to small was mining height, tip-to-face distance, advancing speed, distance of coal seams, surrounding rock strength and support setting load. Later, through similar simulation experiment and numerical simulation, it was clarified that the roof in the process of repeated mining was normal mining, roof deterioration, end face roof leaks, and support crushing four stages. It is pointed out that the tip-to-face distance, support height, and support working condition are the main influencing factors of end face roof control. Based on the above studies, it can be seen that under the condition of close-distance coal seams, due to repeated mining and other factors, the instability mechanism of the end face roof of the lower coal seam is very complex. In the process of lower coal seam mining, it will be affected by many factors such as working face mining height, distance of coal seams, tip-to-face distance, support setting load and so on. The relationship among the influencing factors of stability of end face roof can actually be regarded as a nonlinear relationship.

In the past, as a multivariate nonlinear prediction system for predicting the development law of future events, the artificial neural network has been widely used in various engineering prediction fields^17–19^. There are also many research results based on artificial neural network in the field of coal mine disasters^20–23^. Radial basis function neural network (RBFNN) is widely used in various engineering prediction fields because of its strong approximation ability, fast training speed and simple structure^24,25^. Shu and Gong^26^ used the Latin hypercube sampling method and radial basis function neural network to establish the prediction model of slope safety factor, and used the established prediction model to predict the safety factor. Finally, the reliability index of the slope was calculated by the statistical moment point estimation method. The practical results show that this method is convenient and the results are reliable. At the same time, RBF-ANN as a prediction tool has high accuracy in estimating the effect of asphaltene inhibitors on the reduction of asphaltene precipitation in the petroleum industry^27^. By comparing the Qs index and AUC values obtained by logistic regression, multilayer perceptron artificial neural network and radial basis function artificial neural network, it can be seen that the RBF neural network method has higher accuracy in predicting the probability of rockfalls occurrence caused by earthquakes^28^. Wang et al.^29^ based on the radial basis function neural network prediction method, the water saturation, NMR irreducible water saturation, porosity and permeability are taken as the input layer indexes, and the relative permeability of oil and water is taken as the output layer index. The water cut is calculated by the split flow equation, and it is applied to practice. The prediction effect is good. Gong et al.^30^ selected 12 important influencing factors and the safety evaluation index system for the railway tunnel surrounding rock is constructed. The particle swarm algorithm is used to optimize the RBF neural network training process, and the PCA-IRBF railway tunnel surrounding rock safety prediction and evaluation model is established. Compared with the traditional RBF neural network and PSO-RBF neural network model, the method has a faster convergence speed and smaller error. The accuracy and applicability of the prediction model are proved by practice^31^.

Throughout the literature review, although the radial basis function neural network is widely promoted, there is no research based on this prediction method in the field of coal mine face roof stability. In addition, for different engineering practices, the influence degree of each influencing factor on the stability of the end face roof is different. Therefore, it is necessary to select the representative and universal influencing factors as the input layer index of the neural network, so as to facilitate the construction of the prediction model of end face roof instability.

Therefore, based on the RBF neural network prediction method and through theoretical analysis, this paper establishes the mechanical model of the granular arch structure of the end face roof area, analyzes and summarizes the main influencing factors of the stability of end face roof under repeated mining, and determines the input layer index and output layer index in the neural network structure. By using the functions in MATLAB and through comparative analysis, the rigorous RBF neural network is determined to establish the prediction model. The monitoring and early warning system of instability of end face roof under repeated mining of close-distance coal seams is designed by Python programming language and MYSQL database. Through field application, the practicability of the early warning system is verified. This is of great significance for preventing end face roof disasters under repeated mining.

## RBF neural network

**Figure 1:**
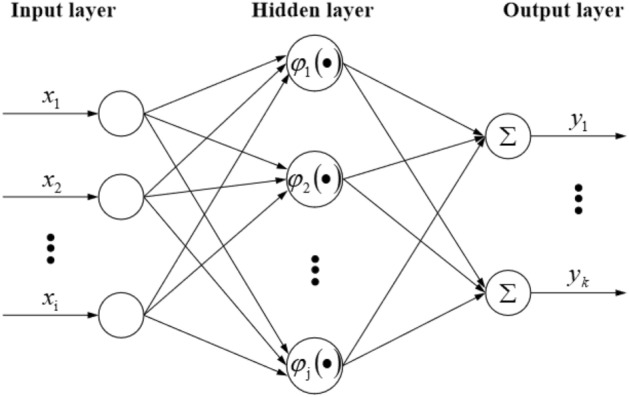
RBF neural network structure.

RBF neural network, as a three-layer feedforward artificial neural network, is widely used in function approximation, signal prediction and other fields^32–35^. Many experts and scholars compared it with BP neural network. The results show that RBF neural network is better than BP neural network in the accuracy of prediction results^36–38^. Since the construction of RBF neural network is a process from nonlinear input to linear output, which is very similar to the relationship between stability of end face roof and its influencing factors under repeated mining of close-distance coal seams, it can be used as a prediction method for stability of end face roof under repeated mining. The structure of RBF neural network includes input layer, hidden layer and output layer, which is shown in Fig. 1. Input layer to hidden layer is a nonlinear mapping process. The hidden layer converts the information input to neurons through a set of symmetric radial basis functions (in most cases, the standard Gaussian function). The process from the hidden layer to the output layer is a linear mapping, which is the process of converting the results of the hidden layer's radial basis function into the results by linear weighted summation.

In the process of building the prediction model, in addition to determining the input layer index and output layer index, the most important is the learning of parameters, including the determination of the center and width of the basis function and the connection weight between the hidden layer and the output layer^39^. In the exploration of artificial neural network method, experts and scholars found that the parameters of hidden layer function of RBF neural network and the connection weights between hidden layer and output layer can be determined by MATLAB software. The research on predicting an event by software has achieved initial results^40^.

## Prediction model based on RBF neural network

### Analysis on influencing factors of stability of end face roof

**Figure 2:**
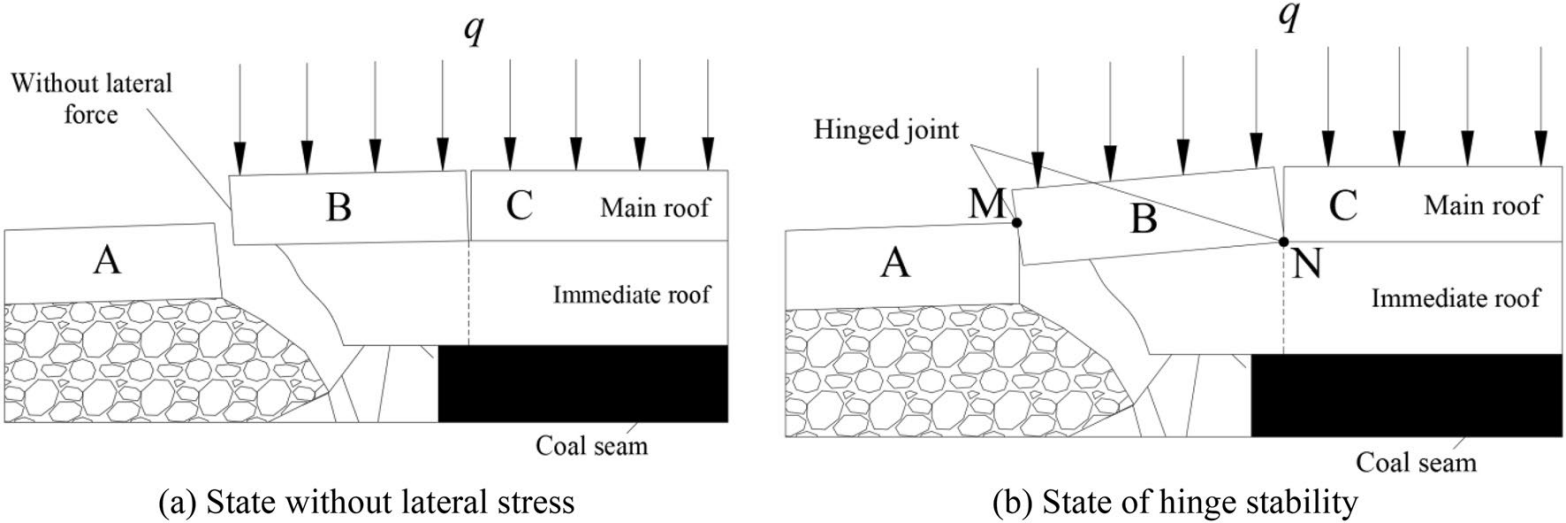
Stability form of key block B on main roof.

In the lower coal seam mining process, the main roof will be broken to form the 'masonry beam' structure. As shown in Fig. 2. With the advance of working face, the block A at the goaf side is fractured and sunk. The block B will also be fractured due to the continuous increase of the exposed area. In general, the fracture position will appear above the advanced working face^41^. Due to the different mining height, block B is either supported by the support and the immediate roof under the 'cantilever beam' structure without the mechanical action of other blocks in the main roof to form a stable structure, or rotates to contact with block A and C to form a temporary hinged stable structure^42^. When the support capacity of the support and the immediate roof is insufficient or the block B continues to rotate, the roof of the mining working face will be in a dangerous state.

Through theoretical analysis, predecessor clarified that the rotation angle of the key block B of the main roof has a great relationship with the crushing degree of the immediate roof in the end face area, and the rotation angle has a great relationship with the mining height of the working face^43^. The strength of the surrounding rock of the roof reflects its bearing capacity of the upper strata and the gangue of the upper coal seam goaf, and also affects the crushing degree of the immediate roof in the end face area. Therefore, mining height of working face and surrounding rock strength of roof can be regarded as important factors affecting the stability of end face roof.

The rotation of the key block B will cause the area close to the coal side below the immediate roof to be squeezed by the main roof and the 'support-coal' structure. With the advance of the working face, the exposed area of the roof strata will continue to increase, and the pressure on the rock in this area will continue to increase. When the pressure exceeds the compressive strength of the rock, the rock in the end face roof area will be destroyed, and then the end face roof will fall. The following will be the mechanical analysis of the broken area of the immediate roof located in the end face roof area.

The rotation of key block B will lead to the crushing of the area near the coal side below the immediate roof due to the extrusion of the main roof and the 'support-coal' structure, which may cause the end face roof caving accident. The following will be the mechanical analysis of the broken area of the immediate roof located in the end face roof area.

**Figure 3:**
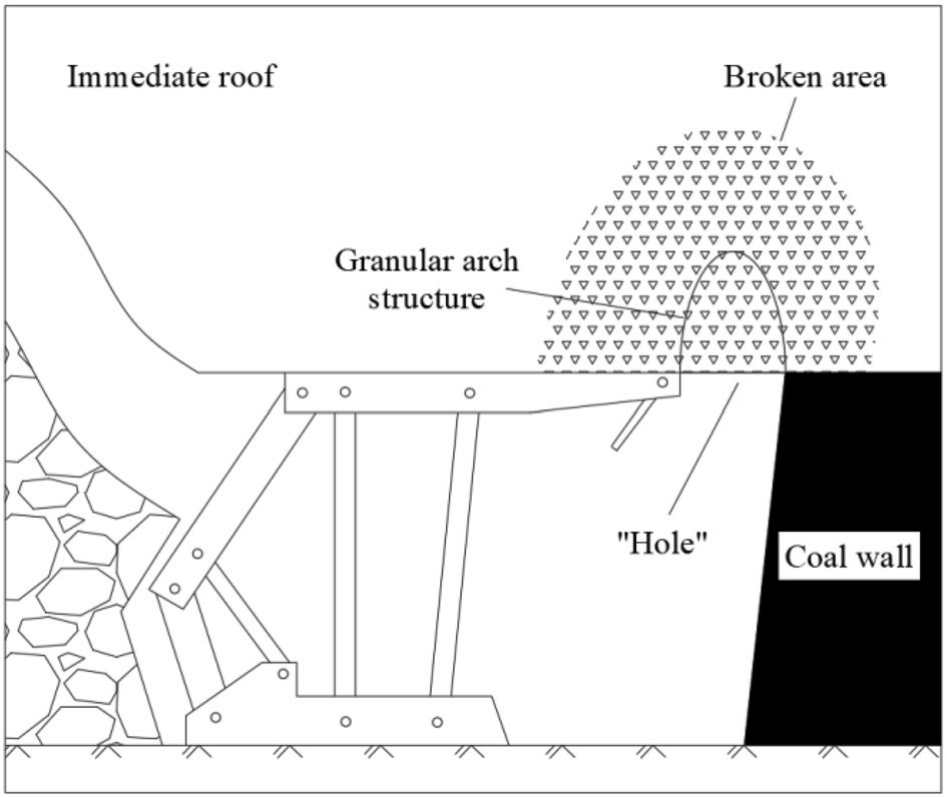
Granular arch structure of end face roof.

First, the area between the coal wall and the end of the support top beam can be assumed to be an infinitely long slot hole, and the broken area at the bottom of the immediate roof above the area can be assumed to be a granular area. Combined with the knowledge of granular mechanics, it can be seen that the mechanical model of granular arch structure will be formed in the granular area above the hole, as shown in Fig. 3.

**Figure 4:**
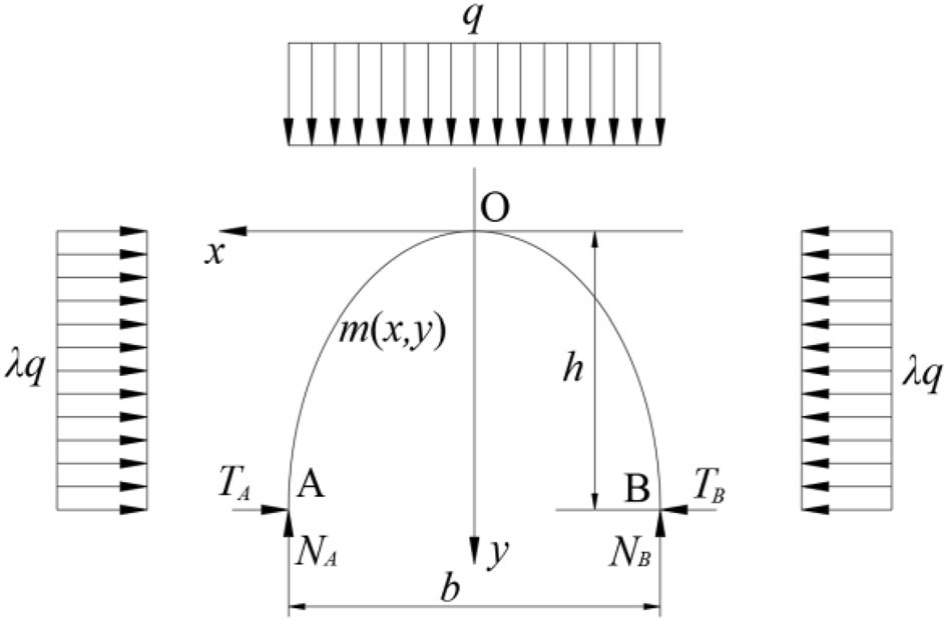
Mechanical model of granular arch structure.

Assuming that the coal body at the coal wall is not destroyed, that is, without considering the rib spalling of the coal wall, the two arch foot points of the granular arch are located on the intersection line between the coal wall and the bottom of the immediate roof and the connection line between the end of the support top beam. To facilitate mechanical analysis, the mechanical model is simplified. Since the spacing between the two arch feet of the bulk arch is not large, the variation of the vertical load distribution along the horizontal direction can be ignored. And the height of the arch is not very large, so it can be assumed that the horizontal load from the immediate roof strata on the side of the coal wall, the horizontal load from the immediate roof strata on the side of the support and the vertical load on the vault are uniformly distributed. As shown in Fig. 4.

In the Fig. 4, *T*_*A*_ and *T*_*B*_ are the horizontal force of the end of the support beam to the arch foot A and the top of the coal wall to the arch foot B, *N*_*A*_ and *N*_*B*_ are the vertical support force of the end of the support beam to the arch foot A and the top of the coal wall to the arch foot B, *q* is the overburden load above the granular arch structure, *λ* is the lateral pressure coefficient inside the immediate roof rock, *h* is the height of the granular arch structure, *b* is the span of the granular arch structure.

Arch line (surface) belongs to the range of free surface of granular material, so the shear force and bending moment at any position along its length direction are zero. The horizontal and vertical forces of the support top beam end on arch foot A are respectively:

1 $$ T_{A} = \frac{{qb^{2} }}{8h} - \frac{\lambda qh}{2} $$

2 $$ N_{A} = \frac{qb}{2} $$

*T*_*A*_ and *N*_*A*_ have the following relationship:

3 $$ T_{A} = f \cdot N_{A} $$

where *f* is the friction coefficient between the end of the support top beam and the granular arch structure.

By substituting Eqs. (1) and (2) into Eq. (3), the relationship between span and height of granular arch can be obtained as follows:

4 $$ b = 2h \cdot \left( {f + \sqrt {f^{2} + \lambda } } \right) $$

Assuming that the tip-to-face distance of roof is *S*, the critical roof caving height is *H*, the horizontal and vertical forces of the support top beam end on the end face roof are *T* and *N*, respectively, and the friction coefficient between the support top beam end and the immediate roof is *f*_0_. When *b* = *S*, *h* = *H,* according to Eqs. (1), (2) and (4), we can obtain:

5 $$ S = 2H \cdot \left( {f_{0} + \sqrt {f_{0}^{2} + \lambda } } \right) $$

6 $$ T = \frac{{f_{0} Sq}}{2} $$

7 $$ N = \frac{Sq}{2} $$

Based on the above equations, it can be seen that the caving height of the end face roof of the mining working face has a great relationship with the tip-to-face distance of roof, and the force of the support top beam end on the horizontal and vertical directions of the end face roof is closely related to the tip-to-face distance of roof. When other conditions are fixed, the larger tip-to-face distance of roof is, the greater the horizontal thrust and vertical support force provided by the support needed to maintain the stability of the end face roof are. Therefore, it can be seen that the tip-to-face distance of roof and support setting load are important factors affecting the stability of end face roof.

In addition, during the mining process of upper coal seam, the internal floor of upper coal seam is affected by mining, which will produce secondary cracks and thus be damaged. This kind of damage may also damage the immediate roof of lower coal seam, so that it has the attribute of granular medium, thus forming the granular arch structure. Therefore, the distance between the lower coal seam and the upper coal seam is also an important factor affecting the stability of the end face roof. At the same time, the advancing speed determines the degree of mining influence. When the advancing speed is greater, the mining influence is greater, and the cracks in the rock mass are more developed, which also greatly affects the stability of the end face roof.

### RBF neural network design

The determination of input layer index is related to the selection of influencing factors of end face roof stability under repeated mining. If the selection of influencing factors is not appropriate, such as selecting factors with small influence as output layer indexes, it is likely to lead to the failure of instability prediction model construction. Therefore, it is necessary to determine the relative importance of each influencing factor. Based on the above analysis of the main influencing factors of the stability of the end face roof, the six influencing factors of mining height, advancing speed, distance of coal seams, tip-to-face distance, surrounding rock strength and support setting load are selected as the input layer indexes in the neural network. Since the stability of end face roof under repeated mining is a qualitative evaluation index, it is necessary to use quantitative indicators to evaluate it. Therefore, the roof subsidence, coal wall deformation and support load can be used as the output layer indexes in the neural network.

Through the above analysis, the six influencing factors index as input layer index, expressed by vector *X*. $X_{r} = \left( {x_{r1} ,x_{r2} ,x_{r3} ,x_{r4} ,x_{r5} ,x_{r6} } \right)^{T}$. Three quantitative evaluation indexes are used as output layer indexes and expressed by vector *Y.* $Y_{r} = \left( {y_{r1} ,y_{r2} ,y_{r3} } \right)^{T}$. Where *r* represents group *r* sample data. The RBF neural network with 6 neurons in input layer and 3 neurons in output layer can be constructed.

### Construction and training of RBF neural network

It is difficult to collect diversified data samples for the data of each index in the input layer under the general situation of a specific project, which makes the final prediction results difficult to be guaranteed in accuracy. At the same time, the scope of application of the designed early warning system will be relatively small. Due to the lack of corresponding measurement equipment in some coal mines, it is difficult to provide reliable data samples for input layer indexes. Therefore, this paper collects the relevant data of multiple coal mines with close-distance coal seams, and obtains some data by numerical simulation based on the general situation of the project, and obtains 100 groups of data as the model samples. Using the randperm function in MATLAB software, 80 groups of data are randomly selected as training data, and the remaining 20 groups of data are used as test data. The specific steps are as follows.

**Figure 5:**
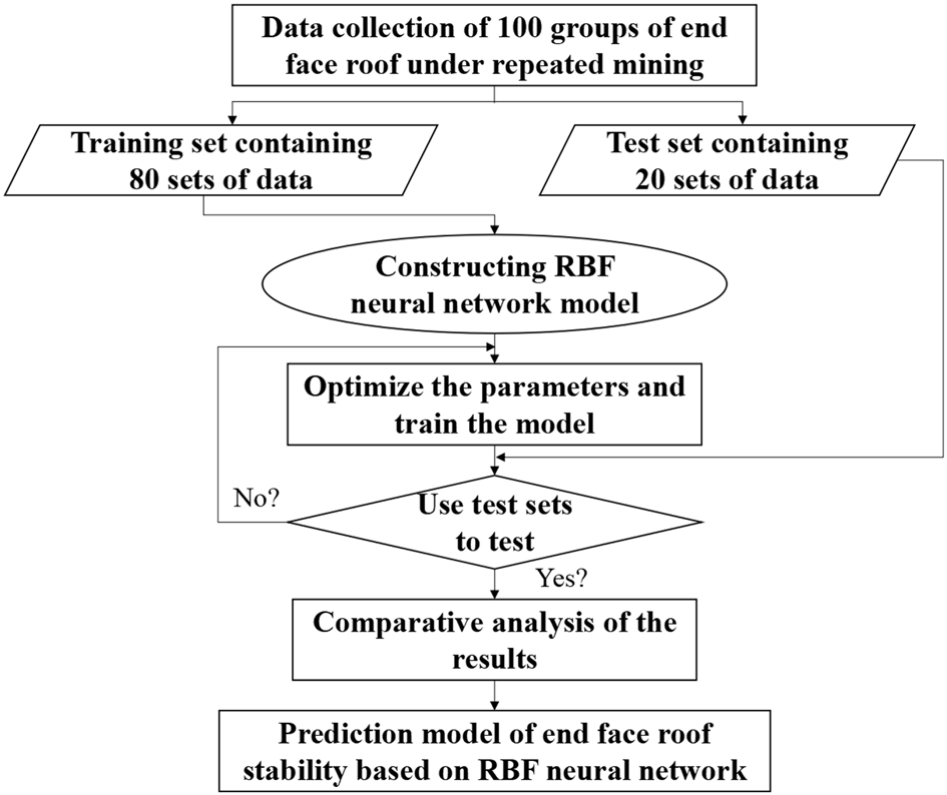
Model logic diagram.

1. Randomly generating training sets with 80 samples and test sets with 20 samples.
2. Using the newrbe function and newrb function of RBF neural network model in the neural network toolbox of MATLAB software, the radial basis function neural network is constructed based on 80 data samples in the training set. The newrbe function is a rigorous RBF neural network model function and the newrb function is a general RBF neural network model function. The newrbe function is similar to the newrb function. In the process of using the newrbe function to construct the neural network, the number of RBF neurons in the network is equal to the number of input samples. The advantage of the newrbe function is that the speed of constructing the neural network is very fast, and an error-free radial basis network can be obtained at one time. However, due to the large scale of the neural network, the data processed at one time is huge. In the process of using the newrb function to construct the neural network, there is no RBF neuron at the beginning. It first starts from the sample with the largest error in the input data, adds a RBF neuron to obtain the corresponding output, and then redesigns the linear layer of the network to gradually reduce the error. In turn, it repeats adding a neuron according to the sample with the largest error in the next occurrence. After the steps before the loop, the error is continuously reduced until the error reaches the specified error performance or the number of neurons reaches the upper limit. The whole model construction process is over. The newrb function is not as fast as the newrbe function in constructing neural network models, but it can obtain smaller neural networks.
3. Using 20 data samples from the test set to simulate the network created.
4. Performance evaluation by relative error, determination coefficient and result comparison.
5. Through the drawing instructions, the broken lines of 20 groups of data output by the rigorous RBF and general RBF neural network models are plotted, including the true value data and predicted value data of roof subsidence, coal wall deformation and support load.
6. The correlation coefficient (R^2^) of the two broken lines is calculated and the results are compared. The logical diagram of the model construction is shown in Fig. 5.

### Analysis of instability prediction model results

**Figure 6:**
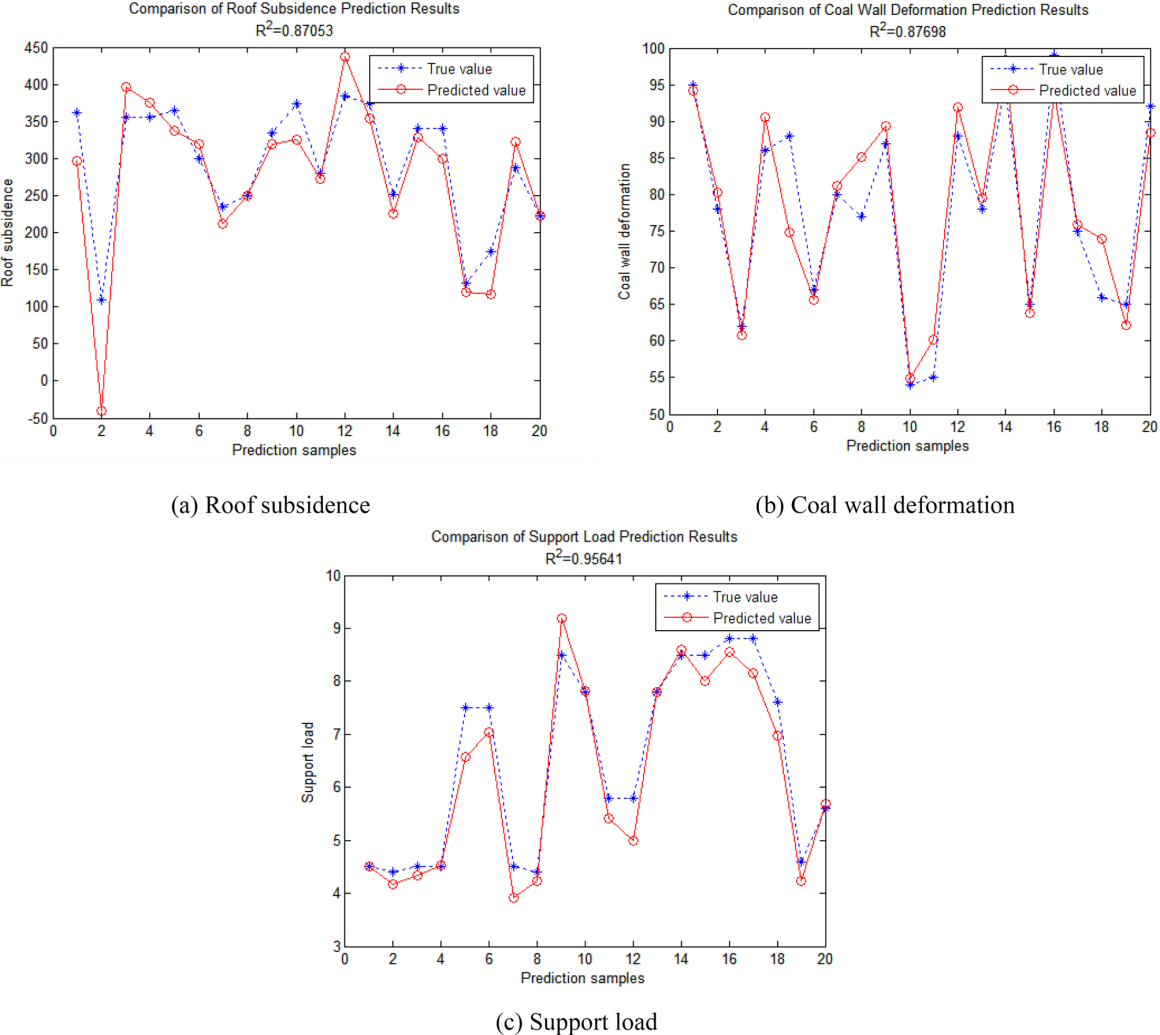
Prediction results of rigorous RBF neural network.

**Figure 7:**
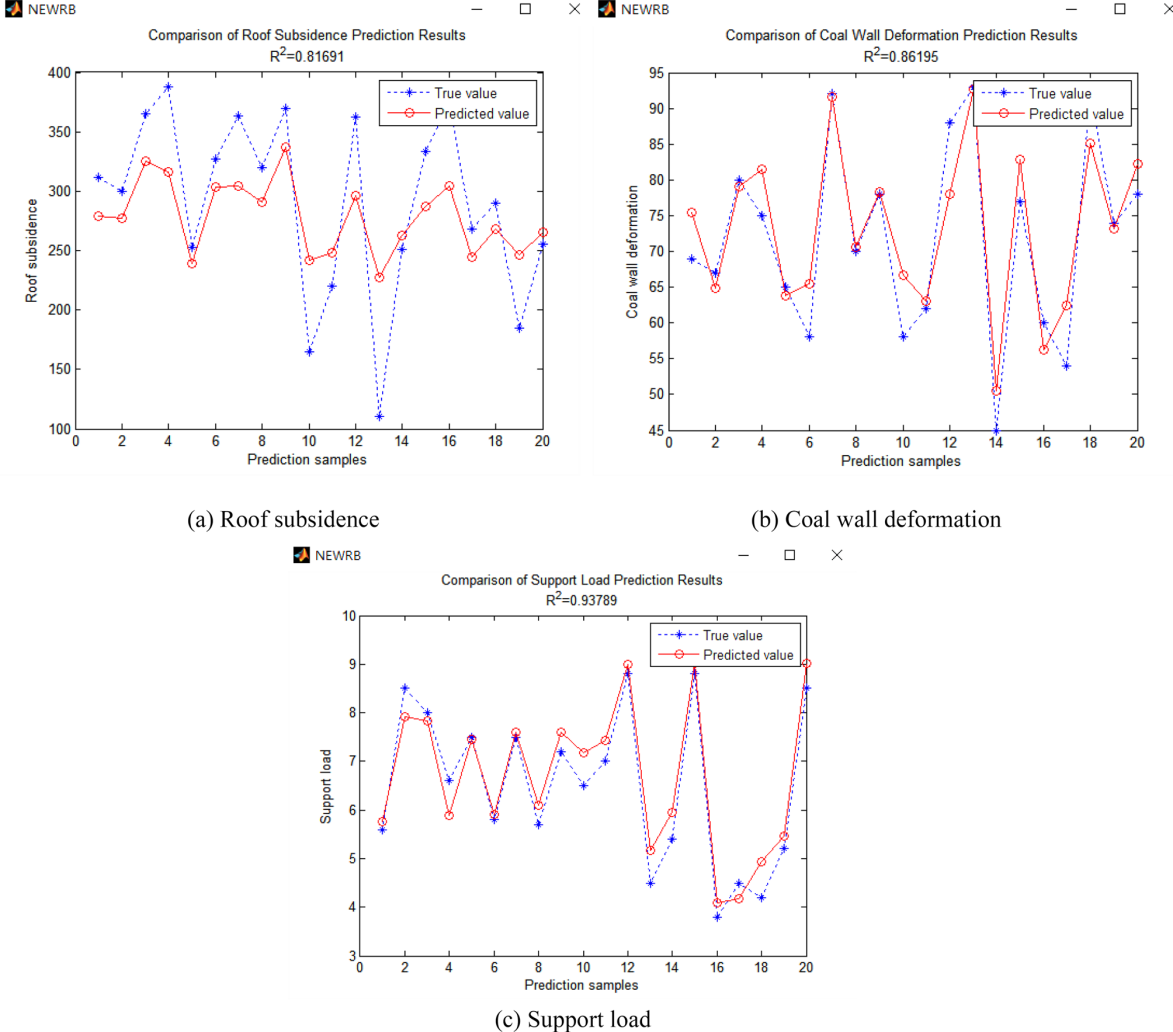
Prediction results of general RBF neural network.

Through the prediction model constructed by two functions in the software, the prediction results of rigorous RBF neural network and general RBF neural network for 20 groups of test data are obtained after drawing instructions, as shown in Figs. 6 and 7. The correlation coefficient (R^2^) of the broken line between the predicted value and the true value obtained by the prediction models constructed by the two different methods is compared, as shown in Table 1.

**Table 1:** Comparison of prediction results between rigorous RBF neural network and general RBF neural network.

	R^2^ of roof subsidence	R^2^ of coal wall deformation	R^2^ of support load
Rigorous RBF neural network	0.87053	0.87698	0.95641
General RBF neural network	0.81691	0.86195	0.93789
R^2^ difference	0.05362	0.01503	0.01852

It can be seen from the results prediction diagram and the above table that the correlation coefficient of the prediction results of each output layer index of the rigorous RBF neural network model is larger than that of the general RBF neural network model. The greater the correlation coefficient means that the predicted value is closer to the real value. The roof subsidence with the largest correlation coefficient difference is above 0.05, which indicates that the prediction model trained by rigorous RBF neural network is more reliable than that trained by general RBF neural network. Therefore, the input data are used to train the rigorous RBF neural network to construct the prediction model of end face roof instability under repeated mining, so as to establish the monitoring and early warning system of instability of end face roof under repeated mining of close-distance coal seams with higher reliability.

## System design

### System design and main interface composition

**Figure 8:**
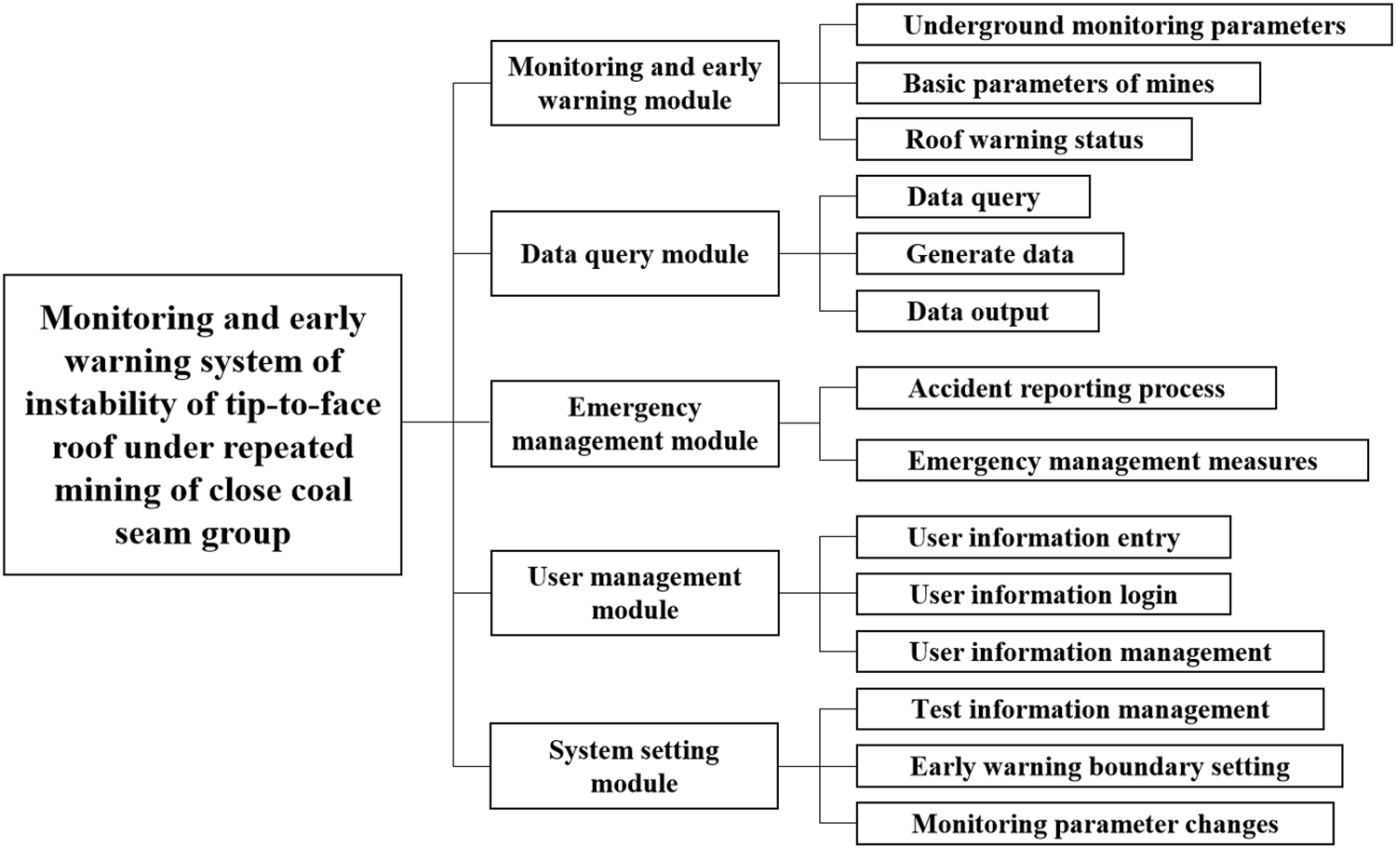
Function module diagram of early warning system.

The monitoring and early warning system of instability of end face roof under repeated mining of close-distance coal seams is based on the data acquisition, transmission and analysis of the three monitoring indexes of the roof subsidence, coal wall deformation and support load. Through the independent design of the early warning system software, the monitoring results are displayed in the form of dynamic curves in real time based on the database data. The monitoring value is compared with the warning threshold value, and then the data of different measuring points are graded. The most important part of the establishment of the early warning system is the design of the early warning system software. Based on the prediction model obtained by rigorous RBF neural network and previous design ideas of early warning system, this paper designs the early warning system by Python programming language and MYSQL database. The system includes five modules: monitoring and early warning, data query, emergency management, user management, and system settings. The monitoring and early warning module is the main module, which can be divided into two independent modules: early warning system and monitoring system, as shown in Fig. 8.

**Figure 9:**
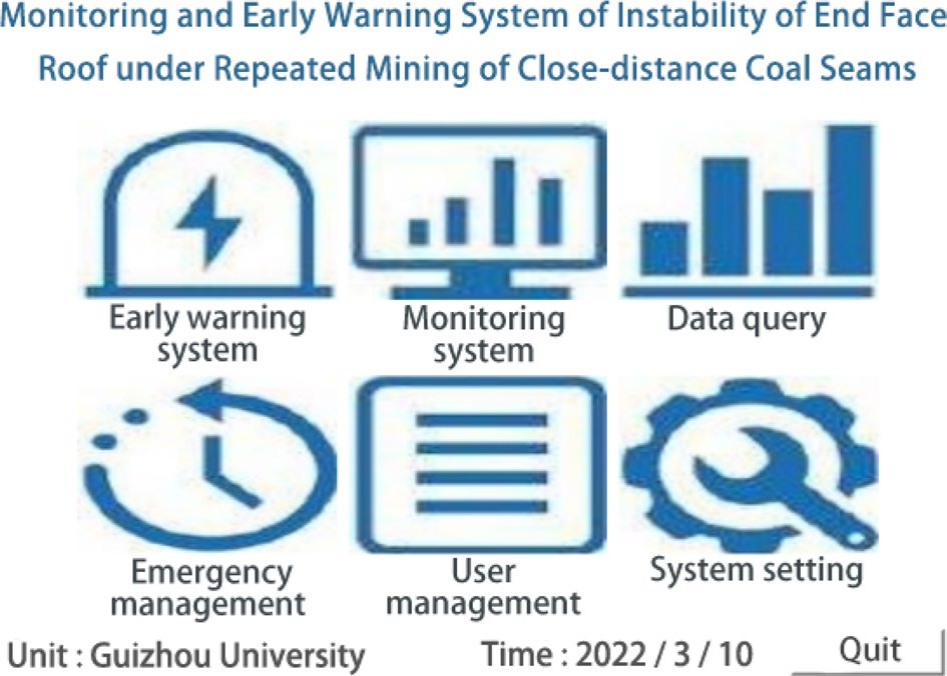
Main interface of early warning system.

Entering this system, the monitoring and early warning system of instability of end face roof under repeated mining of close-distance coal seams is written on the main interface. The interface contains the six modules mentioned above, which correspond to different functions. Clicking on the quit key below the right of the interface can quit the main interface. The basic information such as development unit and login time is written below the main interface. As shown in Fig. 9.

### Module function introduction

1. Early warning system module.
  Through rigorous RBF neural network training out the most suitable prediction model for this system, and then establish early warning system module. This module is the numerical input of parameters such as mining height, advancing speed, distance of coal seams, tip-to-face distance, surrounding rock strength and support setting load. By clicking the upper right corner of the 'RBF neural network prediction' to predict the value of early warning parameters, including roof subsidence, coal wall deformation, support load three characteristic parameters of the early warning value. By comparing with the data monitored on the spot, it is divided into different early warning levels, so as to use the subsequent monitoring system to classify the early warning levels. The warning system module interface is shown in Fig. 10.

**Figure 10:**
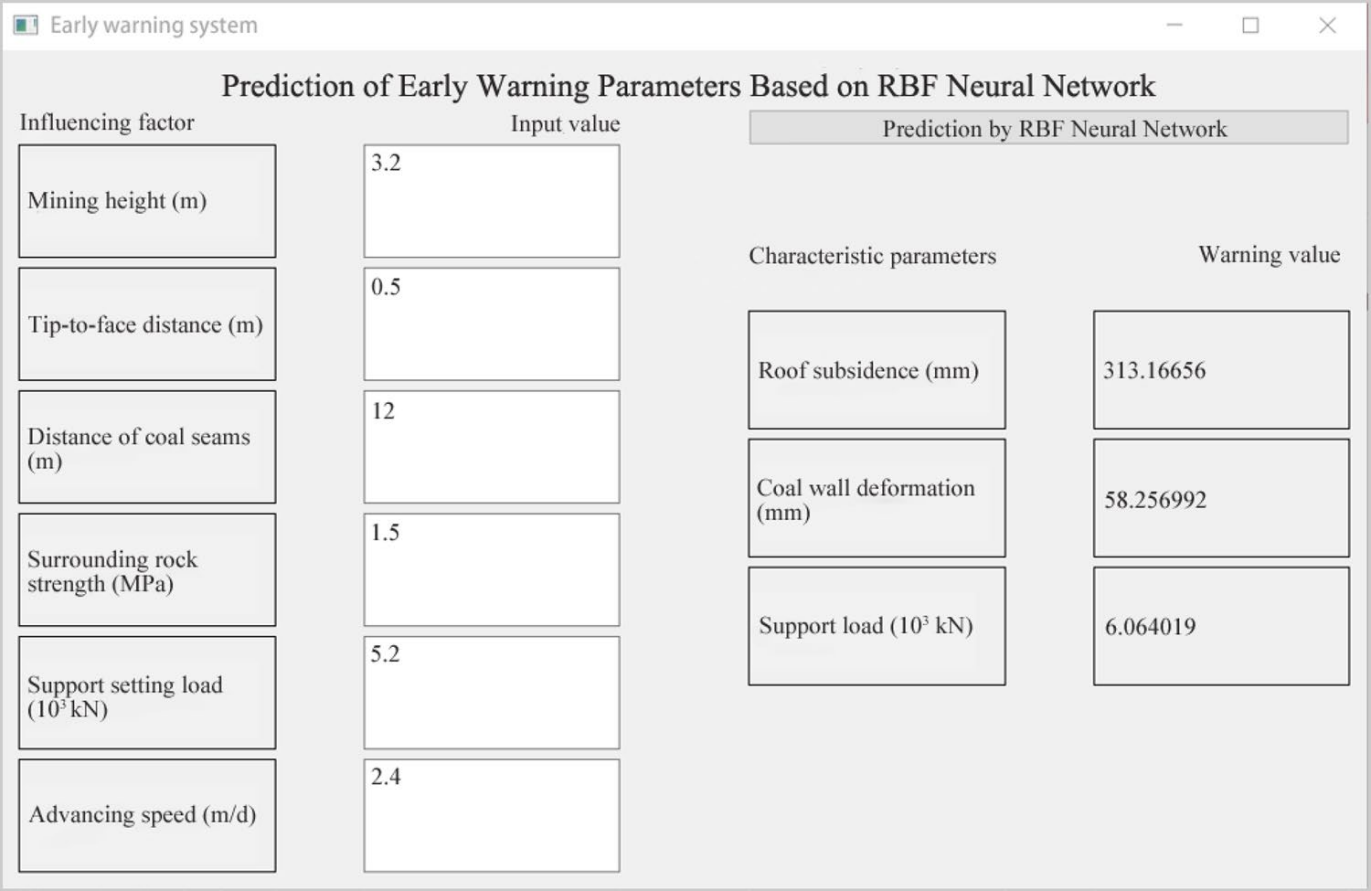
Early warning system interface.
2. Monitoring system module.
  The monitoring system module is based on the data collected by a variety of monitoring equipment in the mine, read the data in the database through the software, execute the software drawing instructions, and draw the real-time monitoring index data curve at each monitoring area. Users can view the dynamic change curve of each index at the number of monitoring area, area and monitoring value of each monitoring area in real time according to the input data. The curve is constantly updated with the continuous change of input data. For the dynamic information of different monitored indexes, users can choose to view it. The state diagram of each index is shown in Fig. 11.

**Figure 11:**
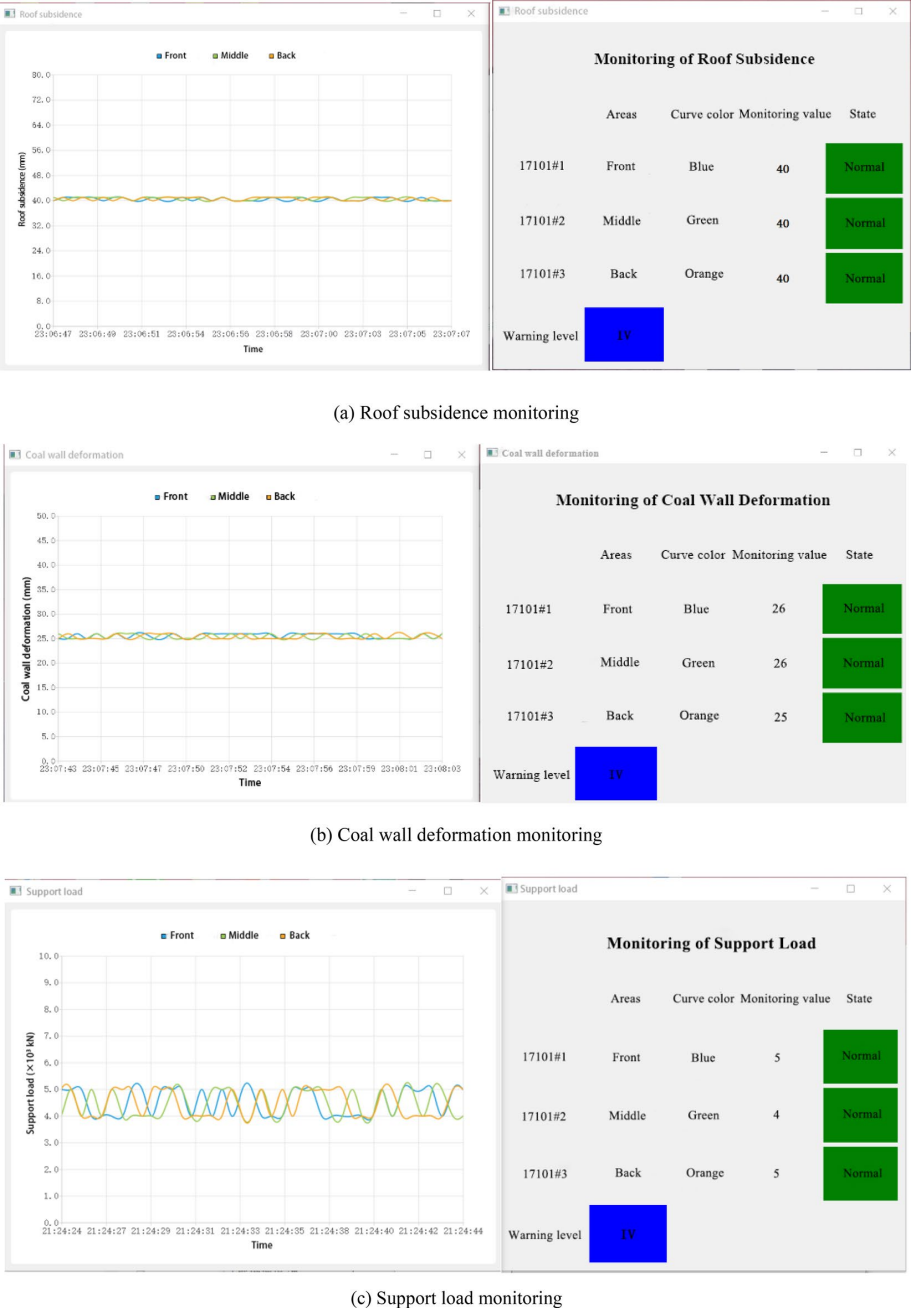
Status of different monitoring indicators.
  At the same time, the situation of mining working face in different area is different. Therefore, in the monitoring system module interface, set the blue, green, orange three color curves are used to represent the changes of monitoring values in front, middle and back of the mining working face. When the monitoring value is less than the warning threshold predicted by the warning system, the monitoring interface shows normal state. When the monitoring value is greater than or equal to the warning threshold value, the monitoring interface will show abnormal state. According to the damage degree of stope face roof disaster and the monitoring status of three monitoring indexes, the early warning level can be divided into four levels, namely IV (ordinary), III (slightly dangerous), II (dangerous), I (special dangerous). In the lower left corner of the interface respectively with blue, yellow, orange, red to represent the four different grades. In ordinary (level IV) cases, the three indexes of roof subsidence, coal wall deformation and support load all show normal state, that is, the monitoring values of the three indicators are all lower than the warning threshold value. At this time, the end face roof is very stable without obvious caving. In slightly dangerous (level III) cases, one of the three monitoring indexes shows abnormal state. At this time, the end face roof is relatively stable, and a small range of caving may occur. In dangerous (level II) cases, two of the three monitoring indexes show abnormal state. At this time, the end face roof is unstable, and a large range of caving may occur. In special dangerous (level I) cases, the three monitoring indexes all show abnormal state. At this time, the end face roof is extremely unstable, and there will be a possibility of large-scale caving at any time. Monitoring personnel should pay attention to the state of measuring points. When one or more indexes exceed the normal value, that is, the condition of end face roof is poor, it should be reported in time, and preventive measures should be made in time through on-site verification, and emergency rescue plan can be started if necessary.
3. Data query module.
  The data query module reads the historical monitoring data stored in the database after selecting the monitoring indexes, the number of monitoring area and time period, and shows the historical monitoring values of the previous specific monitoring index, specific area and specific time for users. This module can provide convenience for later data analysis. At the same time, these historical monitoring data can also be used to provide more data sources for the RBF neural network prediction model and enrich the training samples, so as to optimize the prediction model, make the prediction results closer to the real situation, and enable the system to make accurate analysis. The data query interface is shown in Fig. 12. After setting the monitoring index, measuring point number, query time and other query conditions, click the query, you can see the query below.

**Figure 12:**
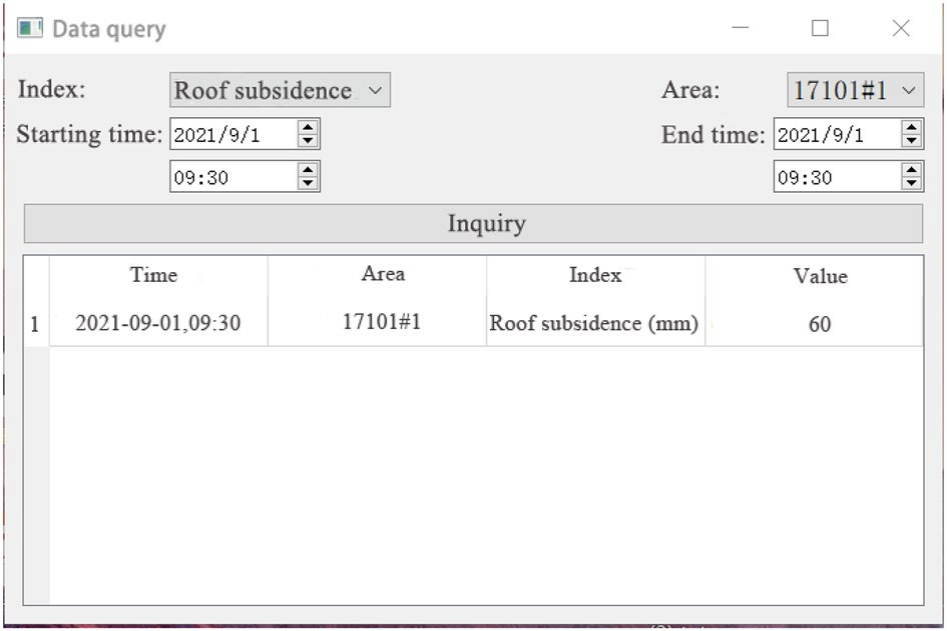
Data query interface.
4. Emergency management module.
  The design of emergency management module is mainly for emergency treatment when an emergency occurs. By starting the emergency plan, so as to quickly and accurately deal with the roof accident, avoid affecting the normal production of coal mine. Under normal circumstances, this module can also be used to access and learn relevant information about coal mine roof disasters. When there is a stope roof disaster accident, the module can facilitate the relevant team leaders to arrange reasonable prevention or rescue measures according to the accident situation in time, and accurately and efficiently deal with the various effects of roof disasters.
5. User management module.
  User management module is mainly designed for the top management personnel of the mine. Through the senior management personnel input the basic information of the relevant staff, so that some staff have the right to login the system. This allows ordinary users to enter the system through input accounts and passwords. In this interface, new user information can be added, users previously recorded can be deleted, and user information recorded can be modified. User management module interface is shown in Fig. 13.

**Figure 13:**
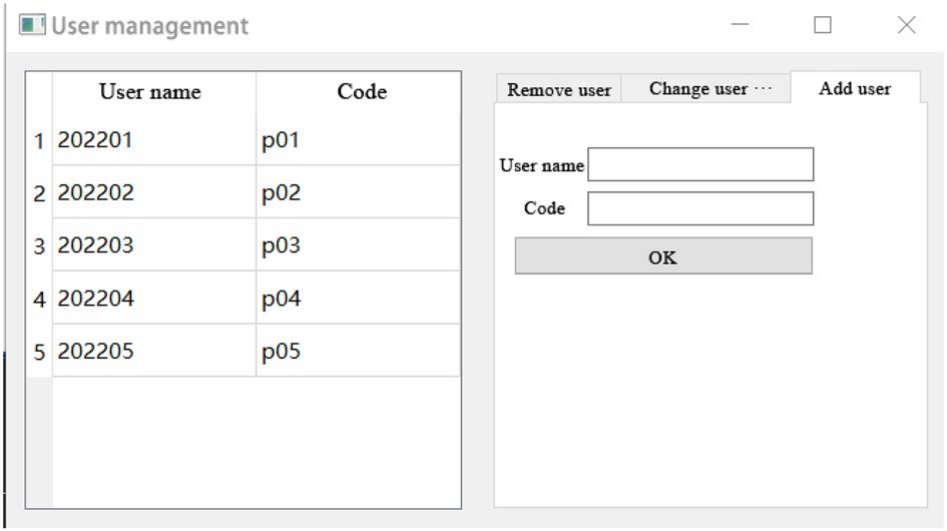
User management interface.
6. System setting module.
  The system setting module is designed for administrators to debug after installing the system. Users can set and modify the system parameters including server IP, port number, link data, and configuration files of different monitoring indexes in detail, so as to meet the requirements of efficient monitoring and early warning for specific mines.

## Field application of early warning system

Taking a mine in Guizhou Province of China as an example, the 15 #, 16 #, 17 # and 18 # coal seams of the mine are all recoverable coal seams, which constitute close-distance coal seams. According to the order from top to bottom, the average thickness of coal seam is 2.0 m, 2.2 m, 3.8 m and 5.0 m respectively, and the average interval between four layers is 5 m, 6 m and 8 m respectively. At present, 15 # and 16 # coal seams have been fully recovered, and 17 # coal seam is being mined at this stage. The 17,101 mining working face is the first working face of the coal seam, and the mining depth is about 550 m. The fully mechanized mining with large mining height is adopted. The mining working face is 180 m long and the advancing length is 1100 m. There have been five large-scale roof caving accidents in the process of mining the working face, and the height of the caving can reach 0.75 m. The caving places are mostly distributed in the unsupported area between the support and the coal wall. Therefore, it is necessary to monitor and warn the end face roof stability of the coal seam working face. Now, the monitoring and early warning system of instability of end face roof under repeated mining of close-distance coal seams is applied to the 17,102 mining working face of the coal seam, and the monitoring and early warning of end face roof are carried out. A set of monitoring equipment is arranged at the front, middle and back ends of the mining working face to monitor the characteristic parameters of the mining working face. The determined six parameters of working face mining height, advancing speed, distance of coal seams, tip-to-face distance, surrounding rock strength and support setting load are input into the early warning system. Through the prediction model, the early warning values of the three characteristic parameters of the working face roof subsidence, coal wall deformation and support load are obtained.

With the advancement of the mining working face, the monitoring equipment collects data in real time and transmits them to the early warning system. The state of each index obtained by monitoring the advance length of each 10 m in the first 100 m after mining of 17,102 mining working face and the warning level obtained by system analysis are recorded and compared with the actual situation. The results are shown in Table 2.

**Table 2:** Comparison of the prediction results of early warning system with the actual situation.

Advancing distance (m)	Monitoring status of indicators			Warning level	Actual situation on site	True or false
	Roof subsidence	Coal wall deformation	Support load			
10	Normal in all	Normal in all	Normal in all	IV	Stable	True
20	Normal in all	Normal in all	Normal in all	IV	Stable	True
30	Normal in all	Normal in all	Normal in all	IV	Stable	True
40	Exist abnormal	Normal in all	Exist abnormal	II	Unstable	True
50	Normal in all	Normal in all	Normal in all	IV	Stable	True
60	Exist abnormal	Normal in all	Normal in all	IV	Stable	True
70	Normal in all	Normal in all	Normal in all	III	Relatively stable	True
80	Normal in all	Exist abnormal	Normal in all	IV	Stable	True
90	Normal in all	Normal in all	Normal in all	IV	Stable	True
100	Normal in all	Normal in all	Exist abnormal	III	Relatively stable	True

Combined with the early warning level of end face roof instability obtained by the early warning system and the actual situation. When the warning levels are IV and III, the end face roof is stable or relatively stable. At this time, the end face roof is in good condition, and there is no caving phenomenon or a small range of caving may occur. When the warning level reaches II, the stability of the end face roof is poor, and it is easy to fall in a large range. In conclusion, the predicted results are consistent with the actual situation. Therefore, the monitoring and early warning system has good practical application effect, which plays an important role in predicting the end face roof disaster under repeated mining of close-distance coal seams.

## Conclusions

1. Through theoretical analysis on the influencing factors of the stability of the end face roof, the mechanical model of the granular arch structure is established, it is determined that the mining height of the working face, the advancing speed, the distance of coal seams, the tip-to-face distance, the strength of the surrounding rock and the support setting the load of the support are the main influencing factors of the end face roof caving. Based on this, the input layer indexes are determined, and the roof subsidence, coal wall deformation and support load are determined as the output layer indexes. The the prediction model of end face roof instability under repeated mining based on RBF neural network is constructed by using the newrbe function and the newrb function in the MATLAB software function toolbox.
2. Through MATLAB software analysis, the prediction results of the model trained by rigorous RBF neural network and general RBF neural network are compared, in order to determine which neural network prediction model prediction effect is better. The results show that the prediction results of the former have a stronger correlation with the real data, so the rigorous RBF neural network prediction model has better accuracy than the general RBF neural network prediction model. Compared with the general RBF neural network prediction model, the correlation coefficient between the predicted value and the real value of the output index of the rigorous RBF neural network prediction model is more than 0.05. Therefore, it is more suitable for the prediction method of end face roof instability under repeated mining, and then the monitoring and early warning system of instability of end face roof under repeated mining of close-distance coal seams is established.
3. The early warning system including five modules of monitoring and early warning, data query, emergency management, user management, and system settings is designed by Python programming language and MYSQL database. The roof conditions of the end face are comprehensively monitored and the possible end face roof accidents can be timely warned. Through field application, it can be seen that the system has good practical value, which is of great significance to prevent the end face roof disaster under repeated mining.

## Data Availability

The data used to support the findings of this research are included within the paper.
